# (Re)integrating radioactive materials and waste into a global sustainable development context

**DOI:** 10.1007/s00411-024-01088-x

**Published:** 2024-08-09

**Authors:** Alexander Wimmers, Fanny Böse, Jasmin Beppler, Pauline Morawe, Maximilian Weber, Christian von Hirschhausen

**Affiliations:** 1 Workgroup for Infrastructure Policy (WIP), Technical University of Berlin (TU Berlin), Straße des 17. Juni 135, 10623 Berlin, Germany; 2 German Institute for Economic Research (DIW Berlin), Mohrenstraße 58, 10117 Berlin, Germany; 3 Federal Office for the Safety of Nuclear Waste Management (BASE), Wegelystraße 8, 10623 Berlin, Germany; 4 TU Berlin, Straße des 17. Juni 135, 10623 Berlin, Germany

**Keywords:** Radioactive waste, Sustainable development goals, Sustainability concepts, Nuclear power

## Abstract

The 17 Sustainable Development Goals (SDGs) of the United Nations, established in 2015, aim to achieve global sustainability by 2030 through the improvement of environmental, social, and economic parameters. However, unlike earlier concepts such as the Agenda 21 of 1992, the SDGs overlook radioactive waste management and related challenges of radiation itself. First, we investigate the historic consideration and unexplained disappearance of radioactive waste in earlier sustainability concepts. Then, we propose amending seven SDGs to address this gap. For SDGs 6 (Clean Water and Sanitation), 14 (Life Below Water), and 15 (Life on Land), new or revised indicators should monitor the release of hazardous materials. SDGs 9 (Industry, Innovation and Infrastructure), 16 (Peace, Justice and Strong Institutions), and 17 (Partnerships for the Goals) require additional targets and indicators to integrate international cooperation and social implications of nuclear facilities' operation. Redefining “hazardous waste” in SDG 12 (Responsible Consumption and Production) and “environmentally sound technologies” in SDG 17 is necessary to encompass radioactive waste. Implementing these changes demands statistical efforts, but the existing monitoring infrastructure, particularly in Europe and North America, can facilitate this. As 2030 approaches, it is crucial to reintroduce radioactive waste management into sustainability agendas, whether within the SDGs themselves or in a subsequent framework.

**Supplementary Information:**

The online version contains supplementary material available at 10.1007/s00411-024-01088-x.

## Introduction

The 17 Sustainable Development Goals (SDGs) mark a global call for action to tackle poverty, pollution, and inequality by 2030 (UN 2015b). Rühm et al. (2023) highlight the impacts of radiological protection measures on and interrelations with a number of SDGs, while Berthiaume (2023a) and Böse et al. (2024a) independently state that the accumulation of radioactive materials and thus its required regulation, monitoring and management are currently underrepresented in global sustainability concepts such as the SDGs. However, this was not always the case. Recognizing the global relevance of potential radiological releases (Lelieveld et al. 2012), the United Nations (UN) published an international agreement on the cooperation for sustainable development in 1992, titled “Agenda 21” (UN 1992). Chapter 22 of this agreement provided several measures to ensure the “safe and environmentally sound management of radioactive wastes” (UN 1992, p. 267). While efforts towards final storage and disposal of radioactive wastes are still in their infancy in most countries (Scheer et al. 2024), and recent pledges of substantial nuclear capacity expansions to supposedly foster decarbonization efforts threaten to further increase radioactive waste volumes (Böse et al. 2024b), the consideration of radioactive wastes and materials is strikingly absent in global sustainability concepts despite earlier emphasis thereon. Thus, in this analysis, we critically assess the current limited role of radioactive materials in the global sustainability context, thereby stressing our concern that human-made radioactive materials should regain their former global relevance via the (re)integration into global sustainability frameworks as major challenges still lie ahead.

The remainder of this work is structured as follows: We first analyze the historic context in which radioactive waste management has played a role in sustainability concepts and show how this role has diminished until today. Then, in “Proposed adaption of SDGs”, we propose several amendments to individual SDGs which should be considered for renewed or updated sustainability concepts beyond 2030. Note that these suggestions are by no means exhaustive, and we therefore express an invitation to further elaborate on this topic and to take this first suggestion as a basis for future considerations. “Discussion” discusses the obtained results and inevitable limitations of our approach, while “Conclusion” concludes.

## Historical context on the disappearance of radioactive materials from sustainability concepts

### Evolution of the SDGs and the consideration of radioactive waste and materials therein

The UN Conference on Environment and Development held in Rio de Janeiro in June 1992 marked a milestone of international cooperation regarding global sustainable development. By forming a “global partnership for sustainable development”, participants aimed at the “fulfilment of basic needs, improved living standards for all, better protected and managed ecosystems, and a safer, more prosperous future” (UN 1992, p. 3). From this, a non-binding treaty called “Agenda 21” was adopted. This framework represented a global consensus and political commitment to implement national strategies and policies. It considered social and economic dimensions, the conservation and management of resources for development, the strengthening of the roles of major groups and the provision of the means for implementation. Notably, Agenda 21 included a detailed chapter on radioactive waste management (UN 1992).

In this chapter, it was recognized that, given the health and safety risks stemming from radioactive materials, the “safe and environmentally sound management of radioactive wastes, including minimization, transportation and disposal, is important” (UN 1992, p. 267). Consequently, nine separate activities were proposed, including activities related to waste management or international and regional cooperation. Examples include the promotion of policies and measures to minimize and limit the accumulation of radioactive waste “where appropriate”, the promotion of “safe” storage and disposal as well as the support of efforts of the International Atomic Energy Agency (IAEA) to “develop and promulgate […] standards or guidelines and codes of practice […] for the safe and environmentally sound management and disposal of radioactive wastes” (UN 1992, pp. 267–68). Activities relating to international and regional cooperation include the goal of replacing the voluntarily moratorium of the London Dumping Convention with a ban on the disposal of low-level radioactive waste at sea (UN 1992).

The implementation of Agenda 21 led to the consideration of the assumptions for standards to protect humans’ and other species’ health from radiation. A series of international conferences in the context of classifying radioactive materials as environmental pollutants, such as the 2003 conference on the “Protection of the Environment from the Effects of Ionizing Radiation” that was held in Stockholm, followed. At this particular conference, the International Commission on Radiological Protection (ICRP) stressed the fact that radiation protection measures had been solely human-focused, and that they should also explicitly demonstrate their sufficiency regarding the protection of the environment and non-human species (IAEA 2005b). A subsequent report by the ICRP (2007) highlighted that global efforts that were required to protect the environment should also include the effects of radiation. Therefore, the ICRP acknowledged the necessity of a systematic framework to assess the relation of exposure and dose for non-human species. The report’s approach proposes reference cases for a wide array of animals and plants and includes respective radiation effects. However, dose limits for environmental protection were not provided.

Other activities suggested by Agenda 21 were partially realized, such as the adoption of a total ban on radioactive waste disposal at sea (with exceptions) for contracting parties following the London Protocol of 1996, for which the IAEA is the internationally responsible authority to provide a database, special permits and definitions (IAEA 2015b; IMO 2006). Nevertheless, the progress is limited. For example, the establishment of highly radioactive waste disposal infrastructures on a national level has not advanced substantially on a global scale: As of today, there is no operational final repository for highly radioactive waste,^1^ and many projects are expected to be operational in a few decades at best (Von Hirschhausen and Wimmers 2023) or postponed indefinitely, such as the Yucca Mountain project in the United States (Wegel et al. 2019).

Despite this limited progress, the focus of global action plans changed over time. With the introduction of the Millenium Development Goals (MDG) in 2000, it shifted towards the reduction of global poverty (UN 2015b, p. 4). Within the objectives of the MDGs, that were to be achieved by 2015, only one goal included the environmental sphere (Goal 7, titled “Ensure Environmental Sustainability”) by including considerations of, e.g., biodiversity loss. The MDGs themselves were very much focused on human needs, e.g., the access to safe drinking water and improvement of the lives of so-called “slum dwellers” (UN 2013, p. 1).

Despite this lack of focus on a global scale, some acknowledgment of the relevance of radioactive materials did occur in parallel to the MDG’s establishment. Today, the UN’s official website includes radioactive waste in the definition of “chemicals and waste” related to SDG 12 (albeit not attributing radioactive wastes to said SDG, see “Acknowledging radioactive waste and materials as hazardous (SDG 12)”), where it briefly recalls main points of Agenda 21 and that radioactive waste was subject of three sessions (5th session in 1997, 7th session in 1999, and 9th session in 2001) of the Commission on Sustainable Development (CSD), with the outcome of stressing the importance of transboundary movement on the World Summit on Sustainable Development in 2002 (UN Undated). However, in preparation of this conference, the CSD published a preparatory document, in which chemical, hazardous waste and radioactive waste were listed explicitly. It was highlighted that the management of radioactive waste had made “good progress” (reduced operational waste volumes by “recycling” and finding of new disposal sites for low-and intermediate level radioactive waste), but progress of finding a repository of high-level waste was “slow” (UN 2001, pp. 6–8). Ten years later, at the 19th session of the CSD in 2011, it was described that “practically all countries generate radioactive waste” (medical, food and crops, industrial) (UN 2011, p. 13). This however did not lead to further concern, despite still existing lack of global progress in waste management. Instead, it was briefly mentioned that national governments should ensure that “appropriate safety measures” were applied to radioactive waste management and that “national strategies, plans and corresponding actions […] [were] developed” (UN 2011, p. 13). The overseeing role of the IAEA was stressed in regard to the “Joint Convention on the Safety of Spent Fuel Management and on the Safety of Radioactive Waste Management” that “is the only legal instrument directly addressing these issues on a global scale” (UN 2011, p. 13).

In the following years, the SDGs were extended from the previous MDGs towards a more expansive approach that recognized that social and economic sustainability can only be achieved if environmental sustainability and the persistence of Earth system processes are ensured (Raworth 2017; Rockström et al. 2009). Consequently, having first been discussed at the Rio + 20 conference in 2012 and implemented in 2015, the SDG framework now consists of a total of 17 individual goals and with a total of 169 targets, each with a set of indicators to measure progress. One overarching goal is to “end poverty and protect the planet” (UN 2023b, p. 1)—with limited success (Leal Filho et al. 2020). While the SDGs now consider several environmental challenges, such as the “reduction of global greenhouse gas emissions” (Target 13.2.2. of SDG 13 (“Climate Action”)) or “the conservation of terrestrial key biodiversity areas” (Target 15.1.2 of SDG 15 (“Life on Land”)) (UN 2023b, p. 3), the role of radioactive materials in the context of sustainability and environmental protection is not acknowledged. For example, SDG 12, titled “Responsible Consumption and Production”, aims to “achieve the environmentally sound management of chemicals and all wastes […] and significantly reduce their release to air, water and soil […]” in its Target 12.4 (One Planet Network 2024). The second of only two indicators, Indicator 12.4.2, is defined as “Hazardous waste generated per capita and proportion of hazardous waste treated, by type of treatment” (UNEP 2021a, p. 11). However, supporting literature relates to the term “hazardous waste” by other types of waste, such as “e-waste” or “chemical waste” and explicitly excludes radioactive waste while building upon earlier regulations such as the EU Waste Framework Directive (European Commission 2010; UNEP 2021a, p. 108).

### UN efforts to develop sustainability indicators for radioactive waste management

**Fig. 1:**
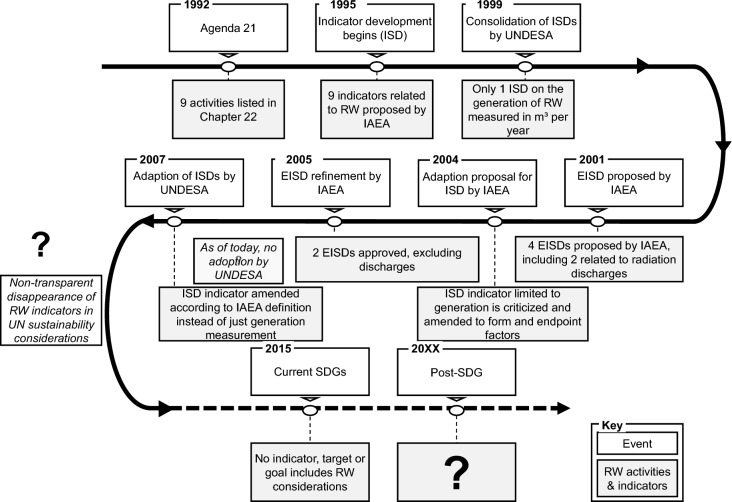
Evolution of the consideration of radioactive materials and waste in UN sustainability indicator development attempts. Sources: Own depiction compiled from (IAEA 2004, 2005a; UN 1992, 2007, 2012, 2015a, 2017; UNDESA 2001; Vera et al. 2005). *EISD* energy-related indicator for sustainable development, *IAEA* International Atomic Energy Agency, *ISD* indicators for sustainable development, *RW* radioactive waste, *SDG* sustainable development goals, *UNDESA* United Nations Department of Economic and Social Affairs

This section provides further detail on the historic development of indicators for radioactive waste and materials in the context of sustainable development and in corresponding frameworks. An overview of the developments is shown in Fig. 1.

Chapter 40 of Agenda 21 called for the development of indicators for sustainable development (UN 1992). In response, the UN Department of Economic and Social Affairs (UNDESA) began to develop indicators for sustainable development (ISD) in 1995 and invited the IAEA to develop one or more for radioactive waste management (IAEA 2004, 2005a). Subsequently, the IAEA’s Division of Nuclear Fuel Cycle and Waste Technology was given the responsibility and by 1999, they had proposed a total of nine indicators for the sustainable management of radioactive waste (ISD-RW) (IAEA 2004).

Over the course of the development period, the total number of indicators from all sectors amounted to 134. Thus, the UNDESA, with support of the UN Statistic Division, consolidated the ISDs to only 58. This reduced set included only one indicator for radioactive waste management, which measured the generation of radioactive waste in m^3^ per year without distinguishing waste types and corresponding activities. The generation of non-radioactive hazardous waste was to be measured by a different indicator. The goal of this single volume-based indicator was to provide an approximation of the hazards stemming from radioactive waste, implying that a growing hazard was directly linked to the accumulation of waste and thus the minimization of waste volumes would contribute to sustainable development (UNDESA 2001).

This consolidation was criticized by the IAEA in two points. Firstly, the consolidated set of 58 indicators by UNDESA had only three indicators related to energy, and thus the IAEA began a long-term program in cooperation with the International Energy Agency (IEA), the UNDESA itself and the IAEA member states to develop a more comprehensive framework of energy-related indicators for sustainable development (EISD) (IAEA 2005b). In the first phase of this project, a set of 41 EISDs were identified. Therein, four indicators were dedicated to radioactive waste. Two aimed at monitoring the release into the environment (“atmospheric radioactive discharges” and “radionuclide discharges into water basins”), and the other two were related to the challenges of radioactive waste management (“generation of radioactive waste” and “quantity of accumulated wastes awaiting disposal”) (Vera et al. 2005, p. 279). The applicability of these indicators was tested by several IAEA members. In parallel, the IAEA refined this set of EISDs together with UNDESA, the IEA, Eurostat, and the European Environment Agency. Based on feedback from the case studies regarding data availability and practicability, the original set of 41 indicators was reduced to 30. This revised set included only the two indicators related to radioactive waste management; the indicators regarding the discharge had been removed (IAEA 2005a; Vera et al. 2005). The two remaining indicators were formulated as the “ratio of solid radioactive waste to units of energy produced” and the “ratio of radioactive waste awaiting disposal to total generated solid radioactive waste” (Vera et al. 2005, p. 282).

Secondly, and in parallel to the development of the EISDs, the IAEA’s Division of Nuclear Fuel Cycle and Waste Technology criticized the single volume-based indicator proposed by UNDESA in 2001 as this would supposedly punish countries that could handle greater amounts of radioactive waste in a “sustainable manner” and apparently used a link between volumes and environmental impacts as “unproven supposition” (IAEA 2004, p. 2). Thus, a different type of indicator was proposed that instead aimed to show the “progress of disposal” (IAEA 2004, p. 2). In this context, sustainability is achieved when “the amount of radioactive waste awaiting disposal is not increasing, the waste is in the final form required for disposal and it is being safely stored” (IAEA 2004, p. 3). Two factors are given to measure the progress of the disposal, both to be measured on a discrete scale from 0 to 50, and then to be added up to a maximum score of 100, which would be the “most sustainable condition” (IAEA 2004, p. 3). The first indicator is the form factor that “indicates the suitability of waste for storage”, and the second indicator is the endpoint factor, that “indicates the status of waste relative to its endpoint” (IAEA 2004, p. 3).^2^ This assessment is to repeated for each waste type, while the IAEA explicitly limits this definition to so-called “managed wastes” and excludes radioactive materials and pollution that is “intentionally released into the environment” (IAEA 2004, p. 3).

However, the development of sustainable indicators remained dynamic. In 2005, the UNDESA’s Division of Sustainable Development decided to review the ISDs to place more emphasis on indicators that are more related to the MDGs (see “Evolution of the SDGs and the consideration of radioactive waste and materials therein”) and to consider the latest developments of indicators. This revision resulted in the third edition of the ISD set, which by then contained a total of 96 indicators with a core set of 50. In this revised version, the IAEA’s 2004 approach of “endpoint and form factors” to measure the progress of radioactive waste disposal was included (UN 2007). The indicators from the EISD set were not adopted.

Ultimately however, the last remaining indicator for radioactive waste management also disappeared from sustainability framework and ISD considerations. In 2012, it was decided at the UN Conference on Sustainable Development (Rio + 20) that a so-called “High-level Political Forum on Sustainable Development” should be established and would replace the CSD of UNDESA (UN 2012). The further development on global indicators was designated to the UN’s Statistical Division (UNSD) via the Inter-Agency and Expert Group adopted at the General Assembly in 2015 (UN 2015a). The global framework of the current SDGs, that were introduced in 2015, includes a total of 231 indicators, with none designated for radioactive waste or radiation (UN 2017).

The above analysis shows that radiation discharge and challenges related to radioactive waste management had been considered in first sustainability indicator and framework considerations. It also shows that for reasons unknown to the authors of this work, the indicators proposed by the IAEA were first reduced to merely volume-based monitoring and then seemingly vanished from any sustainability considerations albeit waste had been continuously accumulating and no (high-level waste) repositories had been built in the meantime. Thus, the proposed adaptions to the SDGs shown in the following section “Proposed adaption of SDGs” are a call for action to reintegrate the measurement of radiation discharges as well as the necessary monitoring of radioactive waste management activities and related challenges into the current SDG framework for the development of a post-2030 sustainability framework.

## Proposed adaption of SDGs

### Overview

The common theme that led to the selection of the seven SDGs for which we propose amendments follows the subsequent rationale: Firstly, the adaption of the SDG framework includes several amendments of existing targets and indicators whose ultimate intentions also apply to radioactive materials, assuming they fall under the category of hazardous materials. Furthermore, new targets and indicators are proposed which are relevant to capture the challenges of radioactive waste management and decommissioning given that most countries operating nuclear power plants are far off from implementing their final storage or disposal solutions (Brunnengräber and Sieveking 2024), and face long-term challenges regarding the decommissioning of said plants (Von Hirschhausen and Wimmers 2023). Thirdly, we discuss the exclusion of radiation and radioactive materials as potential pollutants in the SDG framework. This relates to the definition of “hazardous wastes” and so-called “environmentally sound technologies”, a term that is often substituted with “low carbon”, thus neglecting other pollutants, see “Exclusion of radiation as potential pollutant”.

**Fig. 2:**
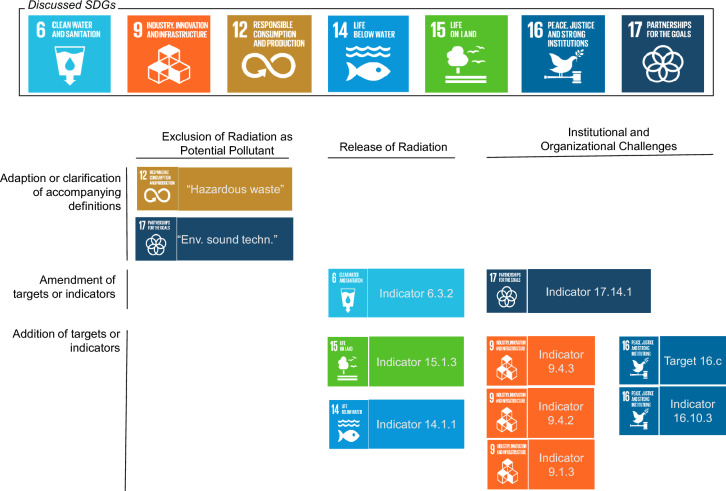
Overview of suggested adaptions to seven SDGs discussed in the following sections. Sources: Own depiction with images taken from https://www.globalgoals.org/goals/ (last accessed 09-05-2024). *Env. Sound techn.* environmentally sound technologies, *SDG* sustainable development goal

Most prominently, the last aspect relates to SDG 7, titled “Clean and Affordable Energy for All”, whose relevance for nuclear power as a low-carbon energy generation source must be critically assessed (Mez 2012; Sovacool 2008), but which shall be omitted here due to its limited scope of energy generation and carbon emissions (UN 2023b). Other SDGs, such as SDG 3 (“Good Health and Wellbeing) or SDG 4 (“Quality Education”) should also be further addressed in future research, albeit their relation to radiation and radioactive materials is not one of environmental release or broader organizational and long-term societal challenges, but more of potential benefits of radiological approaches in medicine and the necessity to ensure the upkeep of global competences in radiological protection (Rühm et al. 2023). Consequently, the here presented amendments are neither collectively exhaustive nor, in acknowledgment of the structure of the SDGs themselves, mutually exclusive. They should be considered as first propositions and require further development and fine-tuning. Figure 2 provides an overview of the proposed amendments to the seven SDGs that are detailed in the following sections. All targets and indicators of the discussed SDGs are provided in the supplementary materials provided in Online Resource 1.

### Exclusion of radiation as potential pollutant

#### Acknowledging radioactive waste and materials as hazardous (SDG 12)

SDG 12, titled “Responsible Consumption and Production” focuses on so-called sustainable production patterns to reduce release of pollutants. Target 12.4 aims to “achieve the environmentally sound management of chemicals and all wastes” and to “reduce their release to air, water and soil” (UN 2024a). The indicators of this Target 12.4 are: 12.4.1, i.e., “Number of parties to international multilateral environmental agreements on hazardous waste, and other chemicals that meet their commitments and obligations in transmitting information as required by each relevant agreement”, and 12.4.2, i.e., “(a) Hazardous waste generated per capita; and (b) proportion of hazardous waste treated, by type of treatment” (UN 2024a). Notably, neither indicator contains any form of measuring scheme to monitor the success of Target 12.4 regarding the release of materials, as they focus on the assessment of generated volumes for specific types of waste, as discussed in “SDG 12: responsible consumption and production”. This also applies to radioactive wastes that are, as mentioned in “Evolution of the SDGs and the consideration of radioactive waste and materials therein”, explicitly excluded from the scope of this SDG (UNEP 2021a).

But as radioactive waste is hazardous, it requires constant and long-term shielding to minimize the release of radiation into the environment, and thus, we propose the amendment of the definition of hazardous waste supplied by (UNEP 2021a) to no longer exclude radioactive wastes and materials. This would then implicitly include radioactive materials in indicators of several SDGs as described in “The release of radioactive materials”.

Regarding the quantifiability of this proposed amendment for radioactive wastes, data availability must improve. While an international agreement on the provision of data on radioactive waste management practices and inventories of various countries exists under the “Joint Convention on the Safety of Spent Fuel Management and on the Safety of Radioactive Waste Management” (IAEA 1997), data availability is limited. Information in the provided reports is often incomplete, outdated and non-standardized regarding, e.g., waste types and units of measurement. For example, reports for the 7th Review Meeting in 2022 overwhelmingly used data from 2019 (IAEA 2023b). Furthermore, the IAEA estimates current global waste inventories in their so-called “Spent Fuel and Radioactive Waste Information System” (SRIS). It becomes apparent that the information is incomplete and very much out of date: at the time of writing, the last update to SRIS had been conducted in 2021, and several country profiles either included no data at all or were outdated by using data from, e.g., 2016 (IAEA 2024).

#### Questioning the term “environmentally sound technologies” (SDG 17)

SDG 17 aims at “strengthen[ing] the means of implementation and revitaliz[ing] the Global Partnership for Sustainable Development” (UN 2024b) and acknowledges the necessity of international cooperation to achieve the ambitious SDG targets, which is reflected by the total of 19 targets. Target 17.7 of SDG 17 is especially relevant in the context of this analysis as it focuses on the “promot[ion of] the development, transfer and diffusion” of so-called “environmentally sound technologies” (UN 2024b) that are also briefly discussed in “SDG 9: industry, innovation and infrastructure” (SDG 9). The only indicator of this target assesses the total amount of funding for so-called developing countries to achieve the target itself.

The UN provides a definition of such “environmentally sound technologies” in a relative manner. Here, technologies are environmentally sound when they “have the potential for significantly improved environmental performance relative to other technologies” (UNSD 2023a, p. 2). The definition explicitly mentions the interchangeability of “environmentally sound” and “low-carbon” (UNSD 2023a, p. 4). While low-carbon technologies are relevant for climate change mitigation (especially for SDGs 7 (Affordable and Clean Energy) and 13 (Climate Action) that are both not discussed in this analysis), the focus on carbon emissions neglects that nuclear power accumulates radioactive materials in form of long-lived hazardous waste and inhibits the risk of spontaneous release of radioactive isotopes into the environment (Präger et al. 2024). If nuclear power were to be defined as such an “environmentally sound technology”, consequentially increased funding would contribute to the expansion of capacities which would in turn inevitably increase radioactive waste volumes as well as at least maintain existing risks of proliferation and accidents. These characteristics have historically led to the exclusion of nuclear power for sustainable development,.e.g., in the Kyoto Protocol of 1997 (IAEA 2017). Thus, we propose to exclude nuclear technology from the definition of environmental sound technologies and thus, activities related to the expansion of nuclear power plants should not fall under the definition of Target 17.7.

Consequentially, promotional activities conducted by the IAEA itself should be more critically discussed, especially regarding its combined role as regulatory and promotional organization (Benz et al. 2022; Katz 2010; Weichselbraun 2016), highlighted, for example, by one-sided publications such as IAEA (2015a) and the continued overly-optimistic prediction of future nuclear capacity expansions (Böse et al. 2024b).

### The release of radioactive materials

Within the SDG framework, the release of hazardous materials is acknowledged in SDGs 6 and 12. Given the current structure of the SDG framework, SDG 12 could be characterized as an overall target because it aims to “significantly reduce” the release of hazardous materials into the air, water and soils (see Chapter 3 of the Supplementary Material). However, no indicator is given to measure the release, instead the focus is placed on hazardous waste generation and its “environmentally sound management”. In “Acknowledging radioactive waste and materials as hazardous (SDG 12)”, we proposed the acknowledgement of radioactive materials as hazardous which would implicitly lead to the adaption of targets and indicators in all other SDGs dedicated to the minimization of the release of hazardous materials. This term is however not always applied, and many targets or indicators list specific chemicals and materials whose release shall be reduced and consequently monitored. For example, SDG 6 partially addresses this issue of the release of anthropogenically generated materials into the marine environment and aims to reduce their release into bodies of water. However, the indicator does not include the release of radionuclides. SDGs 14, and 15 (dedicated to life below water and on land) have not yet included an indicator for the release of hazardous materials. While SDG 14 offers a starting point as it aims to reduce “marine” pollution while referring only to coastal eutrophication^3^ and plastic debris, SDG 15 neglects the issue of the releases of hazardous materials and waste into land-based ecosystems entirely.

In the following, we describe how SDG 6 could be amended to integrate radionuclides. Regarding SDGs 14 and 15, we will propose to acknowledge the release of hazardous materials in general, which would implicitly apply to radioactive materials if SDG 12 is amended according to our proposal in “Acknowledging radioactive waste and materials as hazardous (SDG 12)”. Lastly, we point towards the possibility to integrate release indicators for several pathways into SDG 12.

#### SDG 6: clean water and sanitation

The goal of SDG 6 is to achieve sustainable management of water resources and universal access to sanitation services. For this here analysis, Target 6.3 is relevant. This target aims to “improve water quality by reducing pollution, eliminating dumping and minimizing release of hazardous chemicals and materials” (UN Water 2016, p. 8). In this regard, the term water quality relates to the condition of natural, untreated rivers, lakes and groundwater and the composition thereof in terms of certain substances and chemicals, such as phosphorous, nitrogen, suspended solids, fecal coliform and oxygen concentration (ICWRGC 2023; UN Water 2016; UNSD 2022). The Indicator 6.3.2 “Proportion of bodies of water with good ambient water quality” (UN Water 2016) measures the quality of water with five parameters.

However, radioactive isotopes are not part of these assessments, although they also negatively impact water quality if released and thus should be acknowledged as hazardous materials (see “SDG 12: responsible consumption and production”). Such contaminations can happen through different pathways: Either as a result from spontaneous and uncontrolled releases from nuclear accidents and during power plant operations or via radioactive waste dumping (Gusterson 2017; Livingston and Povinec 2000; Mclaughlin et al. 2012; Rajkhowa et al. 2021). The releases of short-lived isotopes such as tritium are regulated and allowed as long as they remain under authorized discharge limits (IAEA 2010). However, impacts on non-human species remain understudied und monitoring processes do not capture the full picture of contamination, e.g., regarding partial high concentration of tritium in precipitation in the proximity of nuclear power plants (Ferreira et al. 2023; Makhijani 2023).

Consequently, we suggest the amendment of Indicator 6.3.2 “Proportion of bodies of water with good ambient water quality” (UN Water 2016) to extend the estimation of water quality from currently five parameters to at least six by including the assessment of radioactive isotopes undoubtedly stemming from human activities, such as strontium-90, ceasium-137 or tritium, measured, for example, in Bq (Rajkhowa et al. 2021). The measurement and monitoring thereof has been shown to be possible (Berthiaume 2023a). With the proposed amendment of SDG 12 in “Acknowledging radioactive waste and materials as hazardous (SDG 12)”, the term “hazardous materials” would include radioactive waste in the Target 6.3 itself.

#### SDG 14: life below water

SDG 14 relates to the conservation and sustainable use of oceans, seas, and marine resources. Target 14.1 aims to “prevent and significantly reduce marine pollution of all kinds, in particular from land-based activities, including marine debris and nutrient pollution” (UNSD 2024a, p. 14). Target fulfilment is measured by Indicator 14.1.1. that considers two types of pollution: that of coastal eutrophication, and the density of plastic debris in marine environments (UNEP 2021b). Similar to the assessment of SDG 6 above, the lack of consideration of the release of radiological waste and the release of radioactive isotopes from nuclear facilities is apparent.

There has been considerable radioactive waste dumping in the past, in particular from 1946 to 1993 (IAEA 2015b). However, since the 1970s, the problem of marine pollution, explicitly including radioactive materials, was on the agenda of several global inter-governmental conferences, which led to the establishment of the London Convention to prevent marine pollution in 1992; currently having been signed by 87 countries. High-level radioactive waste unsuitable for dumping at sea was first defined by the IAEA (2015b). A voluntary moratorium for the disposal of low-and intermediate waste had already come into action as early as 1985. The Agenda 21 call to replace the moratorium by a ban ultimately led to a global ban on radioactive waste dumping that was established in 1993 (IMO 2006). In this regard, the IAEA requested the contracting parties of the London Convention to provide a global inventory of radioactive materials including dumped waste and actual or potentially release radioactive material into the marine environment from marine accidents and losses. From this, a total activity of marine dumped waste of 8.5 × 10^4^ TBq, including reactors with and without spent fuel (43%) and low-level waste (54%), was estimated (IAEA 2015b). However, releases from “land-based” nuclear facilities (reprocessing) as well as the release of radioactive material from land-based accidents and military activities was excluded, and thus, an additional publication was planned but never realized (IAEA 2015b), although these excluded materials had already been analyzed in literature (Livingston and Povinec 2000). Thus, main anthropogenic radiation sources in oceans result from historic waste dumping and nuclear accidents, and ongoing pollution occurs from the authorized release of reprocessing plants and other facilities. However, as a result of the accident in Fukushima, additional releases of radionuclides have occurred and resulted in concerns about the impacts on marine life and the environment (Schiermeier 2011). Furthermore, the release of waste water is planned to be conducted over the coming decades (22 TBq of tritium per year)^4^ (IAEA 2023a). Depending on respective half-lives, radionuclides entering the marine environment can disperse over longer distances and enter marine food chains via the accumulation in plankton (Inomata and Aoyama 2023; Tan et al. 2023). However, long-term effects are understudied. Ferreira et al. (2024) highlight the need of further research in order to better understand long-term impacts of tritium on marine life and note that future studies should include the presence of emerging stressors such as higher temperatures and plastic debris.

Thus, Indicator 14.1.1 could be amended to include limits of radioactive discharges following IAEA (2010) to limit tritium discharge to 1.5 × 10^14^ Bq per year and other radionuclides to 7.5 × 10^11^ Bq per year, all while taking into account the discharges from the aftermath of accidental releases, such as the Fukushima accident. Consequently, this would necessitate strict monitoring of these materials, as addressed in “SDG 6: clean water and sanitation” (SDG 6) and highlighted by Berthiaume (2023a). It should be noted that such limits may require adaption in the future if knowledge and data, especially about non-human biota, is increased.

Not directly related to the potential release of radioactive materials is the dissipation of heat from nuclear power plants and other heat generating facilities due to necessary cooling, often via external water sources (Herald 2016). Increasing the water temperature in rivers might lead to negative environmental impacts such as the loss of biodiversity (Stewart et al. 2013). The measurement and limitation of thermal pollution could thus also be considered in future SDG targets, applicable to both SDG 6 and 14, but is not included in this here analysis.

#### SDG 15: life on land

SDG 15 aims to protect terrestrial ecosystems, conserve biodiversity and promote sustainable land use practices. This includes measures such as preserving forests, combating desertification, restoring degraded land and protecting endangered species (UN 2023b). However, despite global challenges related to pollution from human activities, such as the spread of released persistent organic pollutants from petrochemical industries (Li et al. 2015) or the industrial air and ground pollution from so-called particulate matter (Mutlu 2020), SDG 15 includes no target that aims at minimizing the release of chemicals or other hazardous materials, unlike Target 6.3 (see “SDG 6: clean water and sanitation”) or Target 14.1 (see “SDG 14: life below water”). Instead, Target 15.1 aims to “ensure the conservation, restoration and sustainable use of terrestrial and inland freshwater ecosystems and their services, in particular forests, wetlands, mountains and drylands” (UNSD 2024b). None of the two indicators is aimed at measuring the release of potentially hazardous materials.

Soil contamination with radioactive materials can happen by the mobilization of naturally occurring radioactive isotopes such as radium-226, thorium-232 and kalium-40 through weathering and erosion but it can also result from human activities such as mining, historic nuclear weapons testing, nuclear accidents or the operation of nuclear power plants (Proshad et al. 2023). Consequently, an Indicator 15.1.3 should be added to Target 15.1 that also considers potential risks from the release of radioactive and other hazardous materials, assuming that radioactive materials have been classified as hazardous as proposed in “Acknowledging radioactive waste and materials as hazardous (SDG 12)”. Such an indicator could read as follows: “Measurement of the release of hazardous materials and chemicals following the definition of Target 12.4 [see “Acknowledging radioactive waste and materials as hazardous (SDG 12)”] into land-based ecosystems via airborne pollution and waste dumping”.

#### SDG 12: responsible consumption and production

Regarding the release of radionuclides into the environment, no specific indicator is provided in SDG 12. As shown above, SDG 6 addresses the release of hazardous material into water while explicitly excluding radionuclides. SDGs 14 and 15 are dedicated to flora and fauna on land and in water but do not include hazardous materials at all. We consequently proposed adaptions to individual targets of theses SDGs.

Given the non-exclusive nature of the SDGs, the proposed adaption of Target 12.4 (see “Acknowledging radioactive waste and materials as hazardous (SDG 12)”) could be expanded to include a set of indicators to not only quantify waste volumes but measure the release of radioisotopes into the biosphere. While infrastructure exists that collects data on many pollutants in so-called pollutant release and transfer registries (PRTR) (OECD 2021), many of these do not include radioisotopes. For example, the Canadian PRTR does not include a single radionuclide, although radionuclides are nationally monitored by authoritative bodies (Berthiaume 2023a; Jackson 2016). Additionally, many research and monitoring data are unavailable to the public, limiting the possibility of independent assessments of releases (Berthiaume 2023b). Notably, Berthiaume (2023a) proposes the integration of PRTR-like data provided by Canadian facilities on the release of radioisotopes into the Canadian PRTR, to provide a more holistic view of pollution that can be incorporated into SDG 12.

In the EU, monitoring of radionuclide release is regulated by Articles 35 and 36 of the Euratom Treaty. EU countries must monitor the level of radioactivity in air, water and soil as well as in food products and thus provide access to a variety of data which are collected in the “Radioactivity Environmental Monitoring Database” (REMdb) (JRC Undated). Furthermore, the European Radiological Data Exchange Platform of the EU’s Joint Research Center was established to represent a standard format for radiological data monitoring in real-time (De Cort et al. 2019). However, such monitoring efforts are not included into the database for monitoring SDGs in the EU; neither for SDG 6 nor SDG 12 (Eurostat 2024). Similar to the Canadian case, these data could surely be integrated into existing PRTRs to provide a more comprehensive and exhaustive overview for the monitoring of the success of the SDGs.

This here analysis aims to provide a first proposal for the integration mainly of radioactive waste and materials into the SDG framework. While the SDGs are of a non-exclusive nature, it could be beneficial to consolidate the proposed amendments to individual indicators on the release of pollutants into different biospheres as proposed in “SDG 6: clean water and sanitation”, “SDG 14: life below water” and “SDG 15: life on land" into a single additional indicator of Target 12.4. The removal of potential redundancies may be addressed in future research.

Thus, future research should address whether SDG 12 should by amended by an indicator or a set of indicators to measure releases of hazardous materials into water, air and soil or if release measurements should be added to the corresponding SDG 6, 14 and 15 as shown above. It might be advantageous to consolidate the releases of radionuclides within Target 12.4. If SDG 12 is chosen to provide indicators, it may require the adaption of the other SDGs to reduce redundancy.

### Institutional and organizational challenges

#### SDG 9: industry, innovation and infrastructure

SDG 9 aims at “building resilient infrastructure, promoting sustainable industrialization and fostering innovation” (UNRIC 2024). This SDG is relevant for this assessment as the operation of nuclear facilities often coincides with the local release of radiation or the contamination and necessary removal of infrastructure after disuse (Hirose and McCauley 2022). Further, the fact that the lack of final disposal repositories of highly radioactive waste in most countries necessitates substantial infrastructure that, due to necessary security measures, is more often than not built from non-sustainable materials (Sovacool 2008), and has often been neglected for future generations to deal with (Brunnengräber and Sieveking 2024), makes this SDG relevant. In the following, we will focus on two targets.

Target 9.1 pursues the establishment of sustainable infrastructure that promotes economic growth or development while supporting human well-being. Measurement indicators are the access to drivable roads for rural populations and the volume of passenger and freight carriers (UNRIC 2024). Both indicators seem to not explicitly include “human well-being”. A major consideration of populations is their sense of security (Edwards et al. 2019). Neles (2019) finds that infrastructure that is perceived as “unsafe”, such as interim storage facilities for highly radioactive waste with pending operation licenses, has an adverse effect on local populations’ sense of security. This claim is to some degree supported by Edwards et al. (2019) who find that populations living in close proximity to nuclear power plants experience a lack of trust in authorities and are more cautious. In contrast however, the operation and subsequent decommissioning of nuclear facilities can positively affect local populations economically and socially (Yamamoto and Greco 2022). Obviously, this assessment is not just applicable to nuclear facilities, and so, consequently, non-sustainable infrastructure, i.e. such that is either related to potential or perceived threats, including nuclear facilities, or such that actively hinders human well-being, such as thermal power plants (Long et al. 2021), should be monitored with an additional Indicator 9.1.3 that could read as follows: “Investments into non-sustainable infrastructure affecting human well-being”. This indicator could monitor planned and ongoing monetary investments into non-sustainable infrastructure, such as the expansion of thermal power plant capacities and impervious surface coverage.

Target 9.4 aims to “to upgrade infrastructure and retrofit industries to make them sustainable, with increased resource-efficiency” (UNRIC 2024). However, the term “resources” is not necessarily limited to only natural or physical resources; it can also be extended to financial resources, especially regarding the relevance of the efficient and responsible utilization thereof (Mulholland et al. 2018; UNECE 2020). As the SDGs aim for long-term sustainability (extending beyond their target date of 2030), the responsible usage of financial resources should also extend beyond that timeframe and take future generations into account. In the context of nuclear materials, the relevance of this is obvious when considering the substantial financial burdens that are being placed upon future generations through historical and ongoing neglect of necessary actions towards, for example, the establishment of final repositories for highly radioactive nuclear waste (Brunnengräber and Sieveking 2024; Von Hirschhausen and Wimmers 2023). As today’s investments and resource allocation affect future generations (Schulze et al. 1981), this consideration should be added as a new Indicator 9.4.2 that could read “Percentage of financially sustainable resources used in the context of intergenerational decisions”. The exact definition of such financially sustainable assets exceeds the scope of this work, but could include considerations of discount and inflation rates, as proposed by Schulze et al. (1981), as well as precautions for potential financial short-falls, often neglected today (Brunnengräber and Denk 2024).

The modernization of existing infrastructure and industry, as proclaimed by Target 9.4, will undoubtedly socio-economically affect local populations, either via the establishment of new industries (Pearman and Starr 2019) or the closure thereof (Brauers et al. 2020; Yamamoto and Greco 2022). Further, the clean-up or continued (necessary) operation of existing potentially non-sustainable infrastructure requires well-trained workforces, which is explicitly relevant to nuclear facilities such as interim waste storage facilities and to-be-decommissioned power plants and could lead to positive socio-economic effects (IAEA 2020; Rühm et al. 2023). Returning to Agenda 21 (see “UN efforts to develop sustainability indicators for radioactive waste management”), one finds that the necessity to develop human resources “to strengthen radioactive waste management infrastructures” was acknowledged as early as 1992 (UN 1992, p. 269), but has since been removed or at least substantially retracted from the public realm. We thus propose another Indicator 9.4.3 to monitor the socio-economic development in affected regions to counteract negative or foster positive socio-economic developments. As job tasks will change over time, this indicator could read “Wages added or lost by industry and infrastructure modernization”.

#### SDG 16: peace, justice and strong institutions

The goals of SDG 16 are to foster the development of peaceful and inclusive societies that contribute to sustainability, provide access to justice to everyone, and establish inclusive institutions at all levels (UN 2023b). In the context of this analysis, Target 16.10 should be further assessed. This target aims to ensure public access to information and protect fundamental freedoms in accordance with national legislation and international agreements. The second indicator of this target, Indicator 16.10.2, that focuses on the access to public information, is based on ten concepts of which the ninth allows the withholding of certain types of information for reasons relating to, for example, national security, privacy, or the management of the economy (UNSD 2021). The UNSD (2021, p. 4) states that such reasons “must be based on narrow, proportionate, necessary and clearly defined limitations” and are applicable where “the [risk of substantial] harm is greater [than] the overall public interest in having access to the information”. However, this definition can be set rather arbitrarily and could thus go against the fundamental principle of transparency^5^ (Finel and Lord 1999). For example, regarding the management of radioactive materials, existing information asymmetries between regulators, the public, and operators can lead to undesirable outcomes, such as significant project delays for waste repository development that, from an outside perspective, seem unnecessary (Von Hirschhausen and Wimmers 2023), especially when transparency on such decisions is low and given reasons seem arbitrary (Röhlig 2023). Given that transparency itself is difficult to define and even more so in the context of concrete measurement of success, we suggest a caveat for the measurement thereof via a third Indicator 16.10.3 for Target 16.10 that could read as follows: “Number of instances in which information is withheld while referring to the definition of “limited exceptions” without providing adequate justification”.

As discussed in “UN efforts to develop sustainability indicators for radioactive waste management”, the Agenda 21 includes a whole chapter dedicated to the “safe and environmentally sound management of radioactive wastes” (UN 1992, p. 267). We thus suggest the implementation of a third target to SDG 16 that should reintroduce the objective of this former goal to “ensure that radioactive wastes are safely managed, transported, stored and disposed of […] within a wider framework of an interactive and integrated approach to radioactive waste management and safety” (UN 1992, p. 267). This new Target 16.c could read “Ensuring the long-term security and safety of radioactive waste management”. In an institutional context, the safety and security of a facility is proven via licensing processes (Smith 2015). Safety, according to the IAEA’s so-called “Fundamental Safety Principles”, encompasses the “protection of people and the environment against radiation risks, and the safety of facilities and activities that give rise to radiation risks” (IAEA 2022, p. 197), while security relates to the threat of “radiological sabotage [, i.e. ,] a deliberate act against a [nuclear facility] that could directly or indirectly endanger public health and safety through exposure to radiation” (Holt and Andrews 2014, p. 1). This is relevant as there exist operating nuclear facilities that have not been licensed, e.g., two interim waste storage facilities in Germany (Becker 2023), that consequentially lack adequate proof of safety and security. We thus propose a first Indicator 16.c.1 that would bring transparency to the issue of non-licensed facilities and could read “Percentage of unlicensed operational nuclear facilities, including waste storage facilities and unlicensed containers”.

Given the substantial risks associated with security issues at nuclear facilities, either via acts of war, as observed in Ukraine (Schneider et al. 2022), or potential acts of terrorism, information on potential risks should be made more transparent by introducing a second Indicator 16.c.2 that would quantify the “Number of criminal or intentional unauthorized attempts to access or conduct destructive actions against nuclear facilities”.

Peaceful and inclusive societies, as stated in the definition of SDG 16, require long-term stability of regulations and institutions. The very long timeframes associated with safe and secure nuclear waste management necessitate such stability over decades to centuries, and from project delays stems the increased focus upon the challenges of long-term interim waste storage (Hardin et al. 2014; Kurniawan et al. 2022; Scheer et al. 2024). For long-term interim storage, a customary assumption is made regarding a storage period ranging from 100 to 300 years (Budelmann et al. 2017), a timeframe that, while marginal in comparison to plans for final waste repositories that can range up to several millennia, remains elusive for humans. Consequently, a strategic framework for the safety of long-term interim storage is also required. The IAEA (2019) recommends the adoption of a mechanism to monitor the national disposal program for radioactive waste. Thus, for the third Indicator 16.c.3, we suggest assessing the “Existence of a valid regulatory framework or action plan for the establishment of long-term interim storage”. It is crucial to note that the overarching, long-term objective of a deep geological repository should not be disregarded in favor of implementing a long-term interim storage solution. This Indicator 16.c.3 is still necessary due to ongoing delays and challenges related to final waste repository site selection and construction projects (Brunnengräber and Sieveking 2024).

#### SDG 17: partnerships for the goals

Target 17.4 of SDG 17, that aims at enhancing policy coherence, might be relevant regarding the management of radioactive materials. Indicator 17.14.1 measures the “[n]umber of countries with mechanisms in place to enhance policy coherence of sustainable development.” This indicator is composed of eight sub-indicators, each quantified on a scale from zero to ten. The second sub-indicator assesses “[l]ong-term considerations in decision-making” (UNEP 2020; UNSD 2023b). This sub-indicator can be linked to the management of radioactive materials, as necessary regulatory frameworks will have to exist for many generations (Pearce 2012). Incorporating so-called “long-term considerations” is imperative for radioactive waste management and should thus be considered in the mentioned sub-indicator. Considering the considerable challenge associated with the establishment or revision of regulatory frameworks within the nuclear energy sector, particularly for emerging nuclear nations in the Global South, it is imperative to deliberate on the adjustment of Target 17.14. Within this context, the challenges range from the inception of regulatory frameworks, as exemplified in the United Arab Emirates, to the harmonization of antiquated legislative measures with international best practices, as evidenced in Bangladesh, Pakistan, and Türkiye (AlKaabi 2022; IAEA 2021). Within the cited sub-indicator, five points are allocated for the inclusion of long-term objectives in national strategies that extend beyond legislative periods (in democratically organized countries), while also considering the interests of future generations. Furthermore, mechanisms promoting intergenerational equity are evaluated. For instance, scoring is contingent upon the presence of mechanisms for the periodic assessment of policy measures to ensure the consideration of unforeseen consequences over time. Another aspect pertains to impact assessment mechanisms that take into account the intergenerational effects of significant infrastructure developments (UNEP 2020). The construction of infrastructure for radioactive waste management can be deemed a significant infrastructure development, see “SDG 9: industry, innovation and infrastructure” (SDG 9). To verify the implementation of the aforementioned second sub-indicator, the United Nations Environmental Program, for example, calls for a nationally recognized government legislation (UNSD 2023b).

In the context of long-term considerations and the interests of future generations, realistic and ambitious timetables that allocate specific timeframes to individual implementation steps are crucial. Furthermore, adherence to these established schedules is of significance. However, the actual implementation of long-term goals is not presently assessed under the sub-indicator. It appears reasonable to incorporate this into the evaluation scale, as the mere formulation of objectives does not guarantee the adherence to long-term goals that consider the interests of future generations. Therefore, the following criterion is recommended: “Mechanisms that monitor the implementation of long-term goals (considering the interests of future generations)” to replace the existing criterion “Other mechanisms of scrutiny or oversight over the possible effects on future generations […]”. This criterion should also be weighted with one point. "Other mechanisms" may vary between different nations, as this mechanism is not precisely defined (UNEP 2020). Thus, the criterion might offer greater flexibility. Alternatively, one could add the above-mentioned criterion and assess the main criterion with four points instead of five.^6^ This way, the overall score would not deviate from the total score of the remaining sub-indicators.

## Discussion

The current SDG framework does not include a single objective or indicator that deals with the challenges of radioactive waste management and releases of radioactive materials into the environment. However, the nuclear industry, including fuel chain activities, power plant operations, decommissioning and radioactive waste management, results in multiple (potential) release pathways for an array of radioisotopes into the air, water, and soil.

Consequentially, this analysis provides a first proposition to adapt the SDG framework in preparation of a follow-up framework beyond 2030. These adaptions reintroduce the challenges of radiation and waste management to the sphere of sustainability concepts. However, an overall picture of global release is complex and thus difficult to grasp. The adaptions have not touched upon the approach to limit the accumulation of radioactive materials and thus to reduce the production of materials and waste. Instead, literature mostly suggests that the continued accumulation of radioactive material should be a part of sustainable development if safe and secure radioactive waste management is ensured and that current occurring releases are authorized and monitored. But recalling the IAEAs definition of the “point of sustainability” (see “UN efforts to develop sustainability indicators for radioactive waste management”), global society is far off from this “sustainable” state, in which radioactive waste volumes are not further increasing and would need to be in their respective final form for disposal, all while being safely stored. Instead, most radioactive wastes are today stored in interim storage facilities awaiting final disposal for which infrastructure mostly does not exist. Thus, the global society still faces the tremendous challenge of final waste disposal while the risks of spontaneous release remain as radioactive wastes and materials continue to accumulate or are at least not reduced.

However, our analysis is by far not exhaustive. For example, it does not consider the interrelations between radioactive wastes and their potential application in nuclear weapons. The Russian attack on Ukraine has resurfaced the long subdued possibility and risks of nuclear fallout which would have social but also global environmental implications (Schneider et al. 2022; Xia et al. 2022). Further, as mentioned in “Overview”, the considerations of nuclear power and radiological hazards might also have implications for SDGs that we did not explicitly analyze, such as SDGs 7 and 13, as these relate more to the low-carbon nature of nuclear power plants, and not the release of radiation. Notably, SDG 13 should be assessed in future research regarding the inclusion of non-CO_2_-related pollutants. Also neglected is Target 12.c that aims to minimize the amount fossil-fuel subsidies via market distortions, which might also be relevant to nuclear power (Bah 2023). This should also be addressed in future research.

There have been earlier suggestions to include issues related to nuclear power into the SDG framework. For example, in 2014, the German Advisory Council on Global Change (WBGU) already proposed to halt the production of fissile material (enriched uranium-235 and plutonium-239) by 2070 (WBGU 2014). They argue that cumulative stocks of radioactive materials (fissile materials such as enriched uranium and plutonium with long half-lives) determine the scale of risks from radiation and potential for weapons production and are persistent in the environment once released. They propose adding targets to a non-specified SDG to stop the production of nuclear fuels for use in weapons and nuclear reactors by 2070. By doing so, they recommend the establishment of the Fissile Material Cut-off treaty. Such a treaty is under consideration to potentially ban the production of fissile material for nuclear weapons and nuclear explosive devices, but has not yet been established (UN 2018). However, future research should address the potential risks stemming from nuclear weapons within the SDG framework.

Furthermore, the UNSD is tasked with performing reviews to refine and improve the SDG framework (UN 2017), but the guiding principles suggest that a new indicator is only accepted in exceptional cases to not further contribute to an additional burden to national statistical work (UN 2023a). While our analysis does not consider the necessary steps to implement the suggested measures on a national basis, we did show that some infrastructure already exists that could be easily integrated into the SDG framework, such as the REMdb or several PRTRs, see “SDG 12: responsible consumption and production”.

Finally, we found that the SDG framework has a non-exclusive nature and includes several redundancies. While we attempted to avoid these in our recommendations, inevitable redundancies emerged, especially with the proposed inclusion of radioactive materials into the category of hazardous materials of SDG 12. This should be addressed in future research to ensure efficient data collection and monitoring.

## Conclusion

By 2030, the 17 SDGs introduced by the UN in 2015 aim to create a more sustainable world for all. The goals consider environmental, social, and economic parameters and targets to monitor and finally achieve this ambitious vision. However, in contrast to earlier sustainability concepts such as the Agenda 21 of 1992, the SDG framework neglects issues and challenges related to radioactive waste management and the potential release of radiation that could pose substantial risks to humans and the environment alike.

In this analysis, we showed that despite the historical consideration of the necessity to include radioactive waste management in sustainability concepts, considerations of this topic have fully disappeared from UN sustainability frameworks and indicators without any evident explanation. Consequentially, we suggest the amendment of seven SDGs. Firstly, we suggest the adaption of two substantial definitions, first of that of “hazardous waste” in SDG 12, that, to this day, explicitly excludes radioactive wastes, and the definition of “environmentally sound technologies” of SDG 17, that is interchangeable with “low-carbon”, thus neglecting an array of relevant pollutants. Secondly, for SDGs 6, 14, and 15, we propose new or amended indicators to existing targets to monitor the release of radioisotopes and other potentially hazardous materials into the environment. Finally, for SDGs 9, 16, and 17, we suggest an array of measures, from the introduction of additional targets to the amendment of existing indicators to reincorporate the historical considerations of international cooperation of organizations and the social implications of radioactive waste management as well as the safe operation of nuclear facilities.

We stress that these suggestions are neither mutually exclusive nor collectively exhaustive. As mentioned above, some goals, such as SDG 7 and 13 are explicitly excluded, and should thus be discussed in subsequent research. We acknowledge that the amendments require additional statistical work and effort, but given the historical considerations of radioactive waste management in earlier sustainability concepts, and the current existence of monitoring infrastructure in Europe and North America, the challenges of radiation and radioactive waste management should be reintroduced to sustainability considerations; perhaps not in the SDGs themselves, but in a follow-up concept, as the target year of 2030 is approaching fast.

## Supplementary Information

Below is the link to the electronic supplementary material.

Supplementary file1 (PDF 339 KB)

## Data Availability

No datasets were generated or analysed during the current study.

## Abbreviations

CSD: Commission on Sustainable Development
EISD: Energy-related indicators for sustainable development
ISD: Indicator for sustainable development
IAEA: International Atomic Energy Agency
ICRP: International Commission on Radiological Protection
IEA: International Energy Agency
MDG: Millennium development goals
PRTR: Pollutant Release and Transfer Registry
REMdb: Radioactivity Environmental Monitoring Database
SRIS: Spent fuel and radioactive waste information system
SDG: Sustainable development goals
UN: United Nations
UNDESA: United Nations Department of Economic and Social Affairs
UNSD: United Nations Statistical Division

## Footnotes

1 The Finnish site is expected to begin operations sometime in 2024 (WNN 2023).

2 Each factor consists of four states that each give 0, 10, 25 or 50 points, respectively, depending on their “sustainability”. For example, for the form factor, state F2 (“capability exists to process waste for storage”) gives 10 points, and state F3 (“capability exists to process waste for endpoint condition”) gives 25 points; for the endpoint factor, state E4 yields the maximum of 50 points and is defined as “inventory of waste not placed into its endpoint is not increasing”, while state E2 (“design and site approved”) gives only 10 points (IAEA 2004, p. 3).

3 Eutrophication is the excessive growth of vegetation in bodies of water that leads to reductions of oxygen levels and potentially hazardous consequences for marine fauna (Chislock et al. 2013).

4 Global tritium release in 2020 is estimated to be around 9000 TBq from operating nuclear power plants and 12.000 TBq for the two European reprocessing plants in La Hague and Sellafield (Gwynn et al. 2024).

5 Transparency can be described as the set of legal, political, and institutional frameworks facilitating the accessibility of information regarding the internal attributes of a government and society to stakeholders within and beyond the domestic political framework. Transparency enables external observers to perceive various significant elements, such as comparative capabilities, interests, and motivations (Mitchell 1998; Oye 1985).

6 Main criterion: “The existence of a long-term objectives going beyond the current electoral cycle, and the interests of future generations embedded in the national sustainable development strategy or policy framework is worth 5 points”. The sub-indicators are uniformly assessed with up to ten points each. The distribution of individual points and the number of criteria being scored is not consistent. Therefore, this approach does not conflict with the scaling of the remaining sub-indicators.
