# Supplementary material for: (Re)integrating radioactive materials and waste into a global sustainable development context
Source: Radiat Environ Biophys. 2024 Aug 9;63(4):519–36. doi: 10.1007/s00411-024-01088-x (PMC11588765; doi:10.1007/s00411-024-01088-x)
Supplement: Supplementary file 1 — Supplementary file1 (PDF 339 KB) [file 411_2024_1088_MOESM1_ESM.pdf]

In the following, we include all targets and indicators of the discussed SDGs 6, 9, 12, 14, 15, 16, and 17. All data is taken from the respective official UN informational websites (UN Water 2016; UNRIC 2024; UN 2024a; 2024b; UNSD 2024a; 2024b; 2024c). Amendments and additions suggested in the main text are given in *cursive*.

# 1 SDG 6 Clean Water and Sanitation

| | Targets | Indicators |
|---|---|---|
| 6.1 | By 2030, achieve universal and equitable access to safe and affordable drinking water for all | 6.1.1: Proportion of population using safely managed drinking water services |
| 6.2 | By 2030, achieve access to adequate and equitable sanitation and hygiene for all and end open defecation, paying special attention to the needs of women and girls and those in vulnerable situations | 6.2.1: Proportion of population using<br>A: safely managed sanitation services and<br>B: a hand-washing facility with soap and water |
| 6.3 | By 2030, improve water quality by reducing pollution, eliminating dumping and minimizing release of hazardous chemicals and materials, having the proportion of untreated wastewater and substantially increasing recycling and safe reuse globally | 6.3.1: Proportion of domestic and industrial wastewater flows safely treated<br>6.3.2: Proportion of bodies of water with good ambient water quality<br>*Adding one or more parameters to indicator 6.3.2 to include the measurement of the release of anthropogenically generated radioisotopes strontium-90, ceasium-137, and tritium in Bq.* |
| 6.4 | By 2030, substantially increase water-use efficiency across all sectors and ensure sustainable withdrawals and supply of freshwater to address water scarcity and substantially reduce the number of people suffering from water scarcity | 6.4.1: Change in water-use efficiency over time<br>6.4.2: Level of water stress: freshwater withdrawal as a proportion of available freshwater resources |
| 6.5 | By 2030, implement integrated water resources management at all levels, including through transboundary cooperation as appropriate | 6.5.1: Degree of integrated water resources management |

| | | |
|---|---|---|
| | | 6.5.2: Proportion of transboundary basin area with an operational arrangement for water cooperation |
| 6.6 | By 2020, protect and restore water-related ecosystems, including mountains, forests, wetlands, rivers, aquifers and lakes | 6.1.1: Change in the extent of water-related ecosystems over time |
| 6.a | By 2030, expand international cooperation and capacity-building support to developing countries in water- and sanitation-related activities and programs, including water harvesting, desalination, water efficiency, wastewater treatment, recycling and reuse technologies | 6.a.1: Amount of water- and sanitation-related official development assistance that is part of a government-coordinated spending plan |
| 6.b | Support and strengthen the participation of local communities in improving water and sanitation management | 6.b.1: Proportion of local administrative units with establishes and operational policies and procedures for participation of local communities in water and sanitation management |

# 2 SDG 9 Industry, Innovation, and Infrastructure

| | Target | Indicators |
|---|---|---|
| 9.1 | Develop quality, reliable, sustainable and resilient infrastructure, including regional and transborder infrastructure, to support economic development and human well-being, with a focus on affordable and equitable access for all | 9.1.1: Proportion of the rural population who live within 2 km of an all-season road.<br>9.1.2: Passenger and freight volumes, by mode of transport<br>*Additional indicator 9.1.3: Investments into non-sustainable infrastructure affecting human well-being* |
| 9.2 | Promote inclusive and sustainable industrialization and, by 2030, significantly raise industry's share of employment and gross domestic product, in line with national circumstances, and double its share in least developed countries | 9.2.1: Manufacturing value added as a proportion of GDP and per capita.<br>9.2.2: Manufacturing employment as a proportion of total employment |
| 9.3 | Increase the access of small-scale industrial and other enterprises, in particular in developing countries, to financial services, including affordable credit, and their integration into value chains and markets | 9.3.1: Proportion of small-scale industries in total industry value added.<br>9.3.2: Proportion of small-scale industries with a loan or line of credit |
| 9.4 | By 2030, upgrade infrastructure and retrofit industries to make them sustainable, with increased resource-use efficiency and greater adoption of clean and environmentally sound technologies and industrial processes, with all countries taking action in accordance with their respective capabilities | 9.4.1: CO2 emission per unit of value added.<br>*Additional indicator 9.4.2: Percentage of financially sustainable resources used in the context of intergenerational decisions*<br>*Additional indicator 9.4.3: Wages added or lost by industry and infrastructure modernization* |
| 9.5 | Enhance scientific research, upgrade the technological capabilities of industrial sectors in all countries, in particular developing countries, including, by 2030, encouraging innovation and substantially increasing the number of research and development workers | 9.5.1: Research and development expenditure as a proportion of GDP<br>9.5.2: Researchers (in full-time equivalent) per million inhabitants |

| | | |
|---|---|---|
| | per 1 million people and public and private research and development | |
| 9.a | Facilitate sustainable and resilient infrastructure development in developing countries through enhanced financial, technological and technical support of African countries, least developed countries, landlocked developing countries and small island developing States | 9.a.1: Total official international support (official development assistance plus other official flows) to infrastructure |
| 9.b | Support domestic technology development, research and innovation in developing countries, including by ensuring a conducive policy environment for, inter alia, industrial diversification and value addition to commodities | 9.b.1: Proportion of medium and high-tech industry value added in total value added |
| 9.c | Significantly increase access to information and communications technology and strive to provide universal and affordable access to the Internet in least developed countries by 2020 | 9.c.1: Proportion of population covered by a mobile network, by technology |

# 3 SDG 12 Resposible Consumption and Production

| | Targets | Indicators |
|---|---|---|
| 12.1 | Implement the 10-Year Framework of Programs on Sustainable Consumption and Production Patterns, all countries taking action, with developed countries taking the lead, taking into account the development and capabilities of developing countries | 14.1.1: Number of countries developing, adopting or implementing policy instruments aimed at supporting the shift to sustainable consumption and production |
| 12.2 | By 2030, achieve the sustainable management and efficient use of natural resources | 12.2.1: Material footprint, material footprint per capita, and material footprint per GDP<br><br>12.2.2: Domestic material consumption, domestic material consumption per capita, and domestic material consumption per GDP |
| 12.3 | By 2030, halve per capita global food waste at the retail and consumer levels and reduce food losses along production and supply chains, including post-harvest losses | 12.3.1: (a) Food loss index and (b) food waste index |
| 12.4 | By 2020, achieve the environmentally sound management of chemicals and all wastes throughout their life cycle, in accordance with agreed international frameworks, and significantly reduce their release to air, water and soil in order to minimize their adverse impacts on human health and the environment<br><br>*Adaption of definition of "hazardous wastes" to no longer explicitly exclude radioactive wastes and materials* | 12.4.1: Number of parties to international multilateral environmental agreements on hazardous waste, and other chemicals that meet their commitments and obligations in transmitting information as required by each relevant agreement<br><br>12.4.2: (a) Hazardous waste generated per capita; and (b) proportion of hazardous waste treated, by type of treatment |
| 12.5 | By 2030, substantially reduce waste generation through prevention, reduction, recycling and reuse | 12.5.1: National recycling rate, tons of material recycled |

| | | |
|---|---|---|
| 12.6 | Encourage companies, especially large and transnational companies, to adopt sustainable practices and to integrate sustainability information into their reporting cycle | 12.6.1: Number of companies publishing sustainability reports |
| 12.7 | Promote public procurement practices that are sustainable, in accordance with national policies and priorities | 12.7.1: Number of countries implementing sustainable public procurement policies and action plans |
| 12.8 | By 2030, ensure that people everywhere have the relevant information and awareness for sustainable development and lifestyles in harmony with nature | 12.8.1: Extent to which (i) global citizenship education and (ii) education for sustainable development are mainstreamed in (a) national education policies; (b) curricula; (c) teacher education; and (d) student assessment |
| 12.a | Support developing countries to strengthen their scientific and technological capacity to move towards more sustainable patterns of consumption and production | 12.a.1: Installed renewable energy-generating capacity in developing and developed countries (in watts per capita) |
| 12.b | Develop and implement tools to monitor sustainable development impacts for sustainable tourism that creates jobs and promotes local culture and products | 12.b.1: Implementation of standard accounting tools to monitor the economic and environmental aspects of tourism sustainability |
| 12.c | Rationalize inefficient fossil-fuel subsidies that encourage wasteful consumption by removing market distortions, in accordance with national circumstances, including by restructuring taxation and phasing out those harmful subsidies, where they exist, to reflect their environmental impacts, taking fully into account the specific needs and conditions of developing countries and minimizing the possible adverse impacts on their development in a manner that protects the poor and the affected communities | 12.c.1: Amount of fossil-fuel subsidies (production and consumption) per unit of GDP |

# 4 SDG 14 Life Below Water

| | Targets | Indicators |
|---|---|---|
| 14.1 | By 2025, prevent and significantly reduce marine pollution of all kinds, in particular from land-based activities, including marine debris and nutrient pollution | 14.1.1: (a) Index of coastal eutrophication; and (b) plastic debris density, and (c) *radiation levels limited to 1.5x10$^{14}$ TBq per year for tritium and 7.5x10$^{11}$ TBq per year for other radioisotopes.* |
| 14.2 | By 2020, sustainably manage and protect marine and coastal ecosystems to avoid significant adverse impacts, including by strengthening their resilience, and take action for their restoration in order to achieve healthy and productive oceans | 14.2.1: Number of countries that manage marine areas in an ecosystem-based manner and the fulfillment of target 14.1 |
| 14.3 | Minimize and address the impacts of ocean acidification, including through enhanced scientific cooperation at all levels | 14.3.1: Average marine acidity (pH) measured at agreed suite of representative sampling stations |
| 14.4 | By 2020, effectively regulate harvesting and end overfishing, illegal, unreported and unregulated fishing and destructive fishing practices and implement science-based management plans, in order to restore fish stocks in the shortest time feasible, at least to levels that can produce maximum sustainable yield as determined by their biological characteristics | 14.4.1: Proportion of fish stocks within biologically sustainable levels |
| 14.5 | By 2020, conserve at least 10 per cent of coastal and marine areas, consistent with national and international law and based on the best available scientific information | 14.5.1: Coverage of protected areas in relation to marine areas |
| 14.6 | By 2020, prohibit certain forms of fisheries subsidies which contribute to overcapacity and overfishing, eliminate subsidies that contribute to illegal, unreported and unregulated fishing and refrain from introducing new such | 14.6.1: Degree of implementation of international instruments aiming to combat illegal, unreported and unregulated fishing |

| | | |
|---|---|---|
| | subsidies, recognizing that appropriate and effective special and differential treatment for developing and least developed countries should be an integral part of the World Trade Organization fisheries subsidies negotiation | |
| 14.7 | By 2030, increase the economic benefits to small island developing States and least developed countries from the sustainable use of marine resources, including through sustainable management of fisheries, aquaculture and tourism | 14.7.1: Sustainable fisheries as a proportion of GDP in small island developing States, least developed countries and all countries |
| 14.a | Increase scientific knowledge, develop research capacity and transfer marine technology, taking into account the Intergovernmental Oceanographic Commission Criteria and Guidelines on the Transfer of Marine Technology, in order to improve ocean health and to enhance the contribution of marine biodiversity to the development of developing countries, in particular small island developing States and least developed countries | 14.a.1: Proportion of total research budget allocated to research in the field of marine technology |
| 14.b | Provide access for small-scale artisanal fishers to marine resources and markets | 14.b.1: Degree of application of a legal/ regulatory/ policy/ institutional framework which recognizes and protects access rights for small-scale fisheries |
| 14.c | Enhance the conservation and sustainable use of oceans and their resources by implementing international law as reflected in the United Nations Convention on the Law of the Sea, which provides the legal framework for the conservation and sustainable use of oceans and their resources, as recalled in paragraph 158 of THE FUTURE WE WANT | 14.c. Number of countries making progress in ratifying, accepting and implementing through legal, policy and institutional frameworks, ocean-related instruments that implement international law, as reflected in the United Nations Convention on the Law of the Sea, for the conservation and sustainable use of the oceans and their resources and the fulfillment of target 14.1 |

# 5 SDG 15 Life On Land

| | Targets | Indicators |
|---|---|---|
| 15.1 | By 2020, ensure the conservation, restoration and sustainable use of terrestrial and inland freshwater ecosystems and their services, in particular forests, wetlands, mountains and drylands, in line with obligations under international agreements | 15.1.1: Forest area as a proportion of total land area<br>15.1.2: Proportion of important sites for terrestrial and freshwater biodiversity that are covered by protected areas, by ecosystem type<br>*Addition of indicator 15.1.3: Measurement of the release of hazardous materials and chemicals following the definition of Target 12.4.2 [see Section 3.4.2] into land-based ecosystems via airborne pollution and waste dumping.* |
| 15.2 | By 2020, promote the implementation of sustainable management of all types of forests, halt deforestation, restore degraded forests and substantially increase afforestation and reforestation globally | 15.2.1: Progress towards sustainable forest management |
| 15.3 | By 2030, combat desertification, restore degraded land and soil, including land affected by desertification, drought and floods, and strive to achieve a land degradation-neutral world | 15.3.1: Proportion of land that is degraded over total land area |
| 15.4 | By 2030, ensure the conservation of mountain ecosystems, including their biodiversity, in order to enhance their capacity to provide benefits that are essential for sustainable development | 15.4.1: Coverage by protected areas of important sites for mountain biodiversity<br>15.4.2:<br>a) Mountain Green Cover Index<br>b) proportion of degraded mountain land |
| 15.5 | Take urgent and significant action to reduce the degradation of natural habitats, halt the loss of | 15.5.1: Red List Index |

| | | |
|---|---|---|
| | biodiversity and, by 2020, protect and prevent the extinction of threatened species | |
| 15.6 | Promote fair and equitable sharing of the benefits arising from the utilization of genetic resources and promote appropriate access to such resources, as internationally agreed | 15.6.1: Number of countries that have adopted legislative, administrative and policy frameworks to ensure fair and equitable sharing of benefits |
| 15.7 | Take urgent action to end poaching and trafficking of protected species of flora and fauna and address both demand and supply of illegal wildlife products | 15.7.1: Proportion of traded wildlife that was poached or illicitly trafficked |
| 15.8 | By 2020, introduce measures to prevent the introduction and significantly reduce the impact of invasive alien species on land and water ecosystems and control or eradicate the priority species | 15.8.1: Proportion of countries adopting relevant national legislation and adequately resourcing the prevention or control of invasive alien species |
| 15.9 | By 2020, integrate ecosystem and biodiversity values into national and local planning, development processes, poverty reduction strategies and accounts | 15.9.1: a) Number of countries that have established national targets in accordance with or similar to Aichi Biodiversity Target 2 of the Strategic Plan for Biodiversity 2011–2020 in their national biodiversity strategy and action plans and the progress reported towards these targets; and<br>b) integration of biodiversity into national accounting and reporting systems, defined as implementation of the System of Environmental-Economic Accounting |
| 15.a | Mobilize and significantly increase financial resources from all sources to conserve and sustainably use biodiversity and ecosystems | 15.a.1: (a) Official development assistance on conservation and sustainable use of biodiversity; and (b) revenue generated and finance mobilized from biodiversity-relevant economic instruments |

| | | |
|---|---|---|
| 15.b | Mobilize significant resources from all sources and at all levels to finance sustainable forest management and provide adequate incentives to developing countries to advance such management, including for conservation and reforestation | (a) Official development assistance on conservation and sustainable use of biodiversity; and<br>b) revenue generated and finance mobilized from biodiversity-relevant economic instruments |
| 15.c | Enhance global support for efforts to combat poaching and trafficking of protected species, including by increasing the capacity of local communities to pursue sustainable livelihood opportunities | 15.c.1:<br>Proportion of traded wildlife that was poached or illicitly trafficked |

# 6 SDG 16 Peace, Justice, and Strong Institutions

| | Targets | Indicators |
|---|---|---|
| 16.1 | Significantly reduce all forms of violence and related death rates everywhere | 16.1.1: Number of victims of intentional homicide per 100,000 population, by sex and age<br>16.1.2: Conflict-related deaths per 100,000 population, by sex, age and cause<br>16.1.3: Proportion of population subjected to (a) physical violence; (b) psychological violence; and (c) sexual violence in the previous 12 months<br>16.1.4: Proportion of population that feel safe walking alone around the area they live after dark |
| 16.2 | End abuse, exploitation, trafficking and all forms of violence against and torture of children | 16.2.1: Proportion of children aged 1-17 years who experienced any physical punishment and/ or psychological aggression by caregivers in the past month<br>16.2.2: Number of victims of human trafficking per 100,000 population, by sex, age and form of exploitation<br>16.2.3: Proportion of young women and men aged 18–29 years who experienced sexual violence by age 18 |
| 16.3 | Promote the rule of law at the national and international levels and ensure equal access to justice for all | 16.3.1: Proportion of victims of violence in the previous 12 months who reported their victimization to competent authorities or other officially recognized conflict resolution mechanisms<br>16.3.2: Unsentenced detainees as a proportion of overall prison population<br>16.3.3: Proportion of the population who have experienced a dispute in the past two years and who accessed a formal or informal dispute resolution mechanism, by type of mechanism |

| | | |
|---|---|---|
| 16.4 | By 2030, significantly reduce illicit financial and arms flows, strengthen the recovery and return of stolen assets and combat all forms of organized crime | 16.4.1: Total value of inward and outward illicit financial flows (in current United States dollars)<br>16.4.2: Proportion of seized, found or surrendered arms whose illicit origin or context has been traced or established by a competent authority in line with international instruments |
| 16.5 | Substantially reduce corruption and bribery in all their forms | 16.5.1: Proportion of persons who had at least one contact with a public official and who paid a bribe to a public official, or were asked for a bribe by those public officials, during the previous 12 months<br>16.5.2: Proportion of businesses that had at least one contact with a public official and that paid a bribe to a public official, or were asked for a bribe by those public officials during the previous 12 months |
| 16.6 | Develop effective, accountable, and transparent institutions at all levels | 16.6.1: Primary government expenditures as a proportion of original approved budget, by sector (or by budget codes or similar)<br>16.6.2: Proportion of population satisfied with their last experience of public services |
| 16.7 | Ensure responsive, inclusive, participatory and representative decision-making at all levels | 16.7.1: Proportions of positions in national and local institutions, including (a) the legislatures; (b) the public service; and (c) the judiciary, compared to national distributions, by sex, age, persons with disabilities and population groups<br>16.7.2: Proportion of population who believe decision-making is inclusive and responsive, by sex, age, disability and population group |
| 16.8 | Broaden and strengthen the participation of developing countries in the institutions of global governance | 16.8.1: Proportion of members and voting rights of developing countries in international organizations |

| | | |
|---|---|---|
| 16.9 | By 2030, provide legal identity for all, including birth registration | 16.9.1: Proportion of children under 5 years of age whose births have been registered with a civil authority, by age |
| 16.10 | Ensure public access to information and protect fundamental freedoms, in accordance with national legislation and international agreements | 16.10.1: Number of verified cases of killing, kidnapping, enforced disappearance, arbitrary detention and torture of journalists, associated media personnel, trade unionists and human rights advocates in the previous 12 months<br>16.10.2: Number of countries that adopt and implement constitutional, statutory and/ or policy guarantees for public access to information<br>*Additional indicator 16.10.3: Number of instances in which `limited exemptions´ cannot be adequately or legally justified* |
| 16.a | Strengthen relevant national institutions, including through international cooperation, for building capacity at all levels, in particular in developing countries, to prevent violence and combat terrorism and crime | 16.a.1: Existence of independent national human rights institutions in compliance with the Paris Principles |
| 16.b | Promote and enforce non-discriminatory laws and policies for sustainable development | 16.b.1: Proportion of population reporting having personally felt discriminated against or harassed in the previous 12 months on the basis of a ground of discrimination prohibited under international human rights law |
| *16.c* | *Additional target: Ensuring safety, security, and long-term safety of radioactive waste management* | *16.c.1: Percentage of operational interim storage sites (for radioactive waste) without license, including waste storage facilities and unlicensed containers*<br>*16.c.2: Number of criminal or intentional unauthorized acts to access or conduct destructive actions against nuclear facilities*<br>*16.c.3: Existence of a valid regulatory framework or action plan for the establishment of long-term interim storage* |

# 7 SDG 17 Partnerships for the Goals

| | Targets | Indicators |
|---|---|---|
| 17.1 | Strengthen domestic resource mobilization, including through international support to developing countries, to improve domestic capacity for tax and other revenue collection | 17.1.1: Total government revenue as a proportion of GDP, by source<br>17.1.2: Proportion of domestic budget funded by domestic taxes |
| 17.2 | Developed countries to implement fully their official development assistance commitments, including the commitment by many developed countries to achieve the target of 0.7 per cent of gross national income for official development assistance (ODA/GNI) to developing countries and 0.15 to 0.20 per cent of ODA/GNI to least developed countries; ODA providers are encouraged to consider setting a target to provide at least 0.20 per cent of ODA/GNI to least developed countries | 17.2.1: Net official development assistance, total and to least developed countries, as a proportion of the Organization for Economic Cooperation and Development (OECD) Development Assistance Committee donors' gross national income (GNI) |
| 17.3 | Mobilize additional financial resources for developing countries from multiple sources | 17.3.1: Additional financial resources mobilized for developing countries from multiple sources<br>17.3.2: Volume of remittances (in United States dollars) as a proportion of total GDP |
| 17.4 | Assist developing countries in attaining long-term debt sustainability through coordinated policies aimed at fostering debt financing, debt relief and debt restructuring, as appropriate, and address the external debt of highly indebted poor countries to reduce debt distress | 17.4.1: Debt service as a proportion of exports of goods and services |
| 17.5 | Adopt and implement investment promotion regimes for least developed countries | 17.5.1: Number of countries that adopt and implement investment promotion regimes for developing countries, including the least developed countries |

| | | |
|---|---|---|
| 17.6 | Enhance North-South, South-South and triangular regional and international cooperation on and access to science, technology and innovation and enhance knowledge-sharing on mutually agreed terms, including through improved coordination among existing mechanisms, in particular at the United Nations level, and through a global technology facilitation mechanism | 17.6.1: Fixed Internet broadband subscriptions per 100 inhabitants, by speed |
| 17.7 | Promote the development, transfer, dissemination and diffusion of environmentally sound technologies to developing countries on favorable terms, including on concessional and preferential terms, as mutually agreed<br>*Adaption of the definition of "environmentally sound technologies" to no longer be interchangeable with "low-carbon" but also consider other pollutants such as radioisotopes* | 17.7.1: Total amount of funding for developing countries to promote the development, transfer, dissemination and diffusion of environmentally sound technologies |
| 17.8 | Fully operationalize the technology bank and science, technology and innovation capacity-building mechanism for least developed countries by 2017 and enhance the use of enabling technology, in particular information and communications technology | 17.8.1: Proportion of individuals using the Internet |
| 17.9 | Enhance international support for implementing effective and targeted capacity-building in developing countries to support national plans to implement all the Sustainable Development Goals, including through North-South, South-South and triangular cooperation | 17.9.1: Dollar value of financial and technical assistance (including through North-South, South-South and triangular cooperation) committed to developing countries |
| 17.10 | Promote a universal, rules-based, open, non-discriminatory and equitable | 17.10.1: Worldwide weighted tariff-average |

| | | |
|---|---|---|
| | multilateral trading system under the World Trade Organization, including through the conclusion of negotiations under its DOHA DEVELOPMENT AGENDA | |
| 17.11 | Significantly increase the exports of developing countries, in particular with a view to doubling the least developed countries' share of global exports by 2020 | 17.11.1: Developing countries' and least developed countries' share of global exports |
| 17.12 | Realize timely implementation of duty-free and quota-free market access on a lasting basis for all least developed countries, consistent with World Trade Organization decisions, including by ensuring that preferential rules of origin applicable to imports from least developed countries are transparent and simple, and contribute to facilitating market access | 17.12.1: Weighted average tariffs faced by developing countries, least developed countries and small island developing States |
| 17.13 | Enhance global macroeconomic stability, including through policy coordination and policy coherence | 17.13.1: Macroeconomic Dashboard |
| 17.14 | Enhance policy coherence for sustainable development | 17.14.1: Number of countries with mechanisms in place to enhance policy coherence of sustainable development<br><br>Sub-indicator: ´2. Long-term considerations in decision-making´<br><br>Allocation of points based on the following aspects:<br><br>• The existence of a long-term objectives going beyond the current electoral cycle, and the interests of future generations embedded in the national sustainable development strategy or policy framework is worth 5 points.<br><br>Additional specific mechanisms (one point each, maximum of five points): |

| | | |
|---|---|---|
| | | • […] A commissioner, council or ombudsperson for future generations;<br>• *Replacement of "Other mechanisms of scrutiny or oversight over the possible effects on future generations of policies or legislation (e.g. auditing mechanisms)" with "Mechanisms that monitor the implementation of long-term goals (considering the interests of future generations)"* |
| 17.15 | Respect each country's policy space and leadership to establish and implement policies for poverty eradication and sustainable development | 17.15.1: Extent of use of country-owned results frameworks and planning tools by providers of development cooperation |
| 17.16 | Enhance the Global Partnership for Sustainable Development, complemented by multi-stakeholder partnerships that mobilize and share knowledge, expertise, technology and financial resources, to support the achievement of the Sustainable Development Goals in all countries, in particular developing countries | 17.16.1: Number of countries reporting progress in multi-stakeholder development effectiveness monitoring frameworks that support the achievement of the sustainable development goals |
| 17.17 | Encourage and promote effective public, public-private and civil society partnerships, building on the experience and resourcing strategies of partnerships | 17.17.1: Amount in United States dollars committed to public-private partnerships for infrastructure |

| | | |
|---|---|---|
| 17.18 | By 2020, enhance capacity-building support to developing countries, including for least developed countries and small island developing States, to increase significantly the availability of high-quality, timely and reliable data disaggregated by income, gender, age, race, ethnicity, migratory status, disability, geographic location and other characteristics relevant in national contexts | 17.18.1: Statistical capacity indicator for Sustainable Development Goal monitoring<br><br>17.18.2: Number of countries that have national statistical legislation that complies with the Fundamental Principles of Official Statistics<br><br>17.18.3: Number of countries with a national statistical plan that is fully funded and under implementation, by source of funding |
| 17.19 | By 2030, build on existing initiatives to develop measurements of progress on sustainable development that complement gross domestic product, and support statistical capacity-building in developing countries | 17.19.1: Dollar value of all resources made available to strengthen statistical capacity in developing countries<br><br>17.19.2: Proportion of countries that (a) have conducted at least one population and housing census in the last 10 years; and (b) have achieved 100 per cent birth registration and 80 per cent death registration |

# References

UN. 2024a. "SDG 12: Ensure sustainable consumption and production patterns." UN Department of Economic and Social Affairs. 2024. https://sdgs.un.org/goals/goal12#targets_and_indicators.

———. 2024b. "SDG 17: Strengthen the means of implementation and revitalize the Global Partnership for Sustainable Development." UN Department of Economic and Social Affairs. 2024. https://sdgs.un.org/goals/goal17#targets_and_indicators.

UN Water. 2016. "Integrated Monitoring Guide for SDG 6. Targets and Global Indicators." United Nations. https://www.unwater.org/sites/default/files/app/uploads/2017/03/SDG-6-targets-and-global-indicators_2016-07-19.pdf.

UNRIC. 2024. "SDG 9." United Nations Regional Information Centre for Western Europe. 2024. https://unric.org/en/sdg-9/.

UNSD. 2024a. "SDG 14 - Conserve and Sustainably Use the Oceans, Seas and Marine Resources for Sustainable Development." 2024. https://sdgs.un.org/goals/goal14.

———. 2024b. "SDG 15 - Protect, Restore and Promote Sustainable Use of Terrestrial Ecosystems, Sustainably Manage Forests, Combat Desertification, and Halt and Reverse Land Degradation and Halt Biodiversity Loss." 2024. https://sdgs.un.org/goals/goal15.

———. 2024c. "SDG 16 - Peace, Justice and Strong Institutions - German Indicators For The UN Sustainable Development Goals." United Nations Statistics Division. February 25, 2024. https://sdg-indikatoren.de/en/16/.
